# Syrup of Hypophosphites

**Published:** 1880-12

**Authors:** 


Syrup of the Hypophosphites.—With an earnest desire at all times to lay before our readers reliable and valuable information in regard to our therapeutic agents it is not always that we can conscienciously do so concerning many of the candidates for professional favor and patronage. It is therefore pleasant to be enabled to speak in terms of high commendation of a preparation that has been long before the profession but which has not heretofore received the attention its merits so well deserve. We refer to the *chemically pure syrup* of the *Hypophosphites*, prepared in accordance with Dr. Chinchills formula, by Dr. J. A. McArthur, of Lynn, Mass. We have used this really elegant preparation, and prescribed it to others with much satisfaction, within the past two or three months, and we do not hesitate to commend it to the attention of the profession as a most valuable agent in the class of diseases in which it has been recommended. In over work of body, or mind, it acts like a charm, in restoring strength and energy to digestion, and the consequent building up of tissue and restoring tone to the system generally.

